# Studies of functional properties of espin 1: Its interaction to actin filaments

**DOI:** 10.3389/fcell.2022.1022096

**Published:** 2022-11-04

**Authors:** Jeong Bin Yang, Kwang Sub Kim, Jiwon Heo, Jeong Min Chung, Hyun Suk Jung

**Affiliations:** ^1^ Division of Chemistry and Biochemistry, College of Natural Sciences, Kangwon National University, Chuncheon, Gangwon, South Korea; ^2^ Department of Biotechnology, The Catholic University of Korea, Bucheon, Gyeonggi, South Korea

**Keywords:** protein-protein interaction, protein purification, Ni-NTA nanogold labeling, transmission election microscopy, espin 1

## Abstract

Actin is a multifunctional biomolecule that forms not only basic structural bodies such as filopodia and lamellipodia, but also large microvilli-like organelles like stereocilia. Actin consists of four sub-domains (S1, S2, S3, and S4), and the “target-binding groove” formed between S1 and S3 is the major binding site for various actin binding proteins. Actin filament dynamics are regulated by numerous actin binding proteins with different mechanisms of actin binding, assembly, and disassembly such as actin severing, branching, and bundling. Ectoplasmic specialization protein 1 (espin 1) is an actin binding and bundling protein that is specifically implicated in the elongation and stabilization of stereocilia as a binding partner with myosin III. However, little is known about the molecular structure, actin bundling, and stabilizing mechanism of espin 1; hence, we investigated the interaction between actin and espin 1 through structural data. In this study, we first purified human espin 1 in an *E. coli* system following a new detergent-free approach and then demonstrated the 2D structure of full-length espin 1 using transmission electron microscopy along with Nickel nitrilotriacetic acid nanogold labeling and 2D averaging using SPIDER. Furthermore, we also determined the espin 1 binding domain of actin using a co-sedimentation assay along with gelsolin and myosin S1. These findings are not only beneficial for understanding the actin binding and bundling mechanism of espin 1, but also shed light on its elongation, stabilization, and tip-localization mechanisms with myosin III. This study thus provides a basis for understanding the molecular structure of espin 1 and can contribute to various hearing-related diseases, such as hearing loss and vestibular dysfunction.

## Introduction

Many actin-binding and bundling proteins such as Arp2/3, gelsolin, and espin are implicated in actin cytoskeleton regulation (Bartles, 2000; Small et al., 2002; Rotty et al., 2013; Pollard, 2016). The espin family has four isoforms 1–4, and are known to play key roles in various organelles such as spermatid-, taste receptor-, cochlear hair-, and vestibular hair-cells (Bartles et al., 1998; Bartles, 2000; Zheng et al., 2000; Sekerková et al., 2003; Naz et al., 2004; Sekerková et al., 2006a). Espin 1, the first identified and largest isoform of the espin family, has been observed in the parallel actin bundles of unique junctional plaques called “ectoplasmic specializations” in Sertoli cells (Bartles et al., 1996; Salles et al., 2009; Merritt et al., 2012). It is denominated as “espin” (ectoplasmic specialization + in) based on its cellular localization. Further studies have revealed an additional three isoforms found in various mechanosensory and chemosensory organelles as actin cytoskeleton regulator proteins that elongate microvilli similarly to parallel actin bundles. Actin bundle elongation is important for hair cell development in vestibular and cochlear cells. “ESPN” mutations in the espin-encoding gene lead to vestibular dysfunction “deafness” (Naz et al., 2004). The mutations lead to shortened stereocilia with reduced stiffness, which causes the symptoms of this autosomal recessive disorder.

**FIGURE 1:**
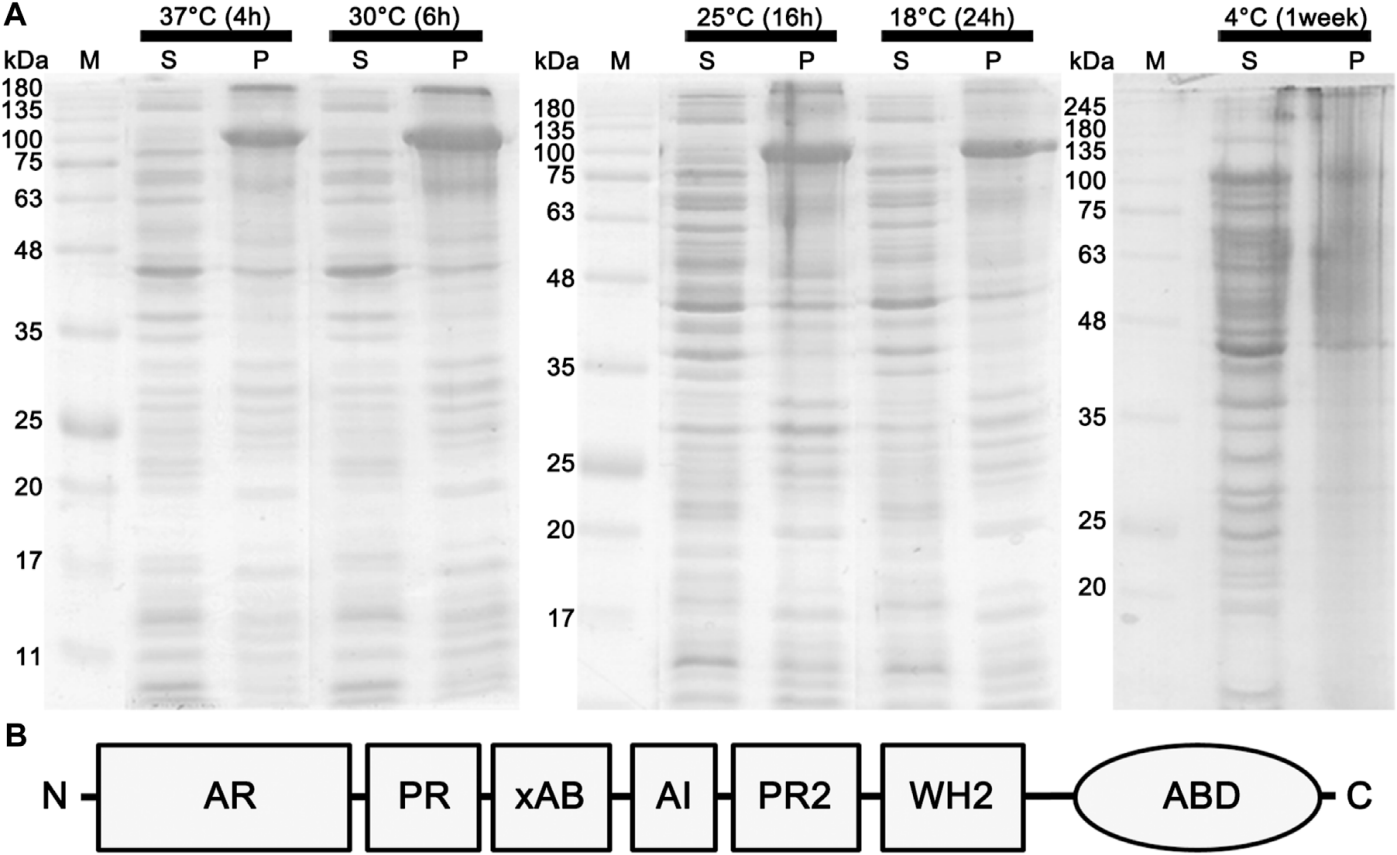
Effects of different temperature conditions on the expression of the full-length espin 1. **(A)** SDS-PAGE analysis demonstrating the expression level of espin 1. Effects of different temperature condition on the expression of the full-length espin 1 were indicated by whether it is soluble (lane S) or insoluble (lane P). The growth conditions, including temperatures and cultivation periods varied ranging from 4°C to 37°C and 4 h to 1 week respectively. M represents the marker. Black arrows point to the expressed espin 1 protein. **(B)** Domain organization of espin 1.

Espin 1 is a candidate target for therapies for vestibular dysfunction, and several studies have been conducted to reveal the functional properties of espin 1 at the molecular level. Its various domains have previously been characterized (Sekerková et al., 2006b); however, the protein-protein interactions leading to actin cross-linking *via* espin 1 remain unclear. Most actin-bundling proteins in stereocilia, including fascin and fimbrin, contain at least two or more actin-binding domains, which function as oligomers to cross-link two or more actin filaments (Matsudaira, 1994; Pollard, 2016). Likewise, the espin 1 monomer contains two F-actin binding domains. In addition, it contains an extra actin binding site (xAB), a C-terminal actin binding domain (ABD), and a G-actin binding domain called the WASP homology 2 (WH2) domain (Chen et al., 1999; Sekerková et al., 2004; Sekerková et al., 2006a) (Figure 1B). A previous study showed that the actin bundling activity espin 1 xAB is blocked by an autosuppressive region termed the auto-inhibitory domain (AI), which binds to the N-terminal ankyrin repeat (AR) of espin 1. It then induces xAB inactivation through steric hindrance. The tail THDI domain of myosin III can reverse this self-inhibition by binding with the N-terminal AR domain of espin 1 and replacing the AI domain. (Liu et al., 2016). In general, two or more free actin-binding domains are required to form actin bundles. Thus, in the case of espin 1, it is theoretically impossible to form a bundle when a binding partner, such as myosin III, is absent because it contains only one free F-actin binding domain at the C-terminus. However, espin 1 can induce the formation of actin bundles and localizes at the tip of stereocilia without its binding partner, myosin III (Loomis et al., 2003). This indicates that espin 1 can bypass its own self-inhibition in the absence of a binding partner. However, there is no clear explanation for the mechanism underlying this phenomenon, and it is only known that purified recombinant espin 1 molecules exist as monomers in solution (Bartles et al., 1998; Chen et al., 1999) to help produce parallel microvilli-like actin bundles. Therefore, to understand the actin-espin 1 interaction accurately at the molecular level, structure-based research is required. This study aims to provide more detailed information on the mechanisms of actin bundling *via* espin 1 using electron microscopy structural analysis techniques.

## Materials and methods

### Purification of recombinant espin 1

The full-length espin 1 gene (GenBank accession No. NG_015866) was inserted downstream of the T7 promoter into the plasmid vector pET-30a (+). The resultant plasmid DNA constructs were transformed into *Escherichia coli* BL21 (DE3) star cell lines, and transformants were selected on LB agar medium (Luria Bertani Broth High Salt; Kisanbio, Seoul, Republic of Korea, Bacto Agar; Bacton, Dickinson and Company, Franklin Lakes, NJ) supplemented with 50 μg/ml kanamycin (Table 1). Transformants were grown in LB medium containing 50 μg/ml kanamycin at various temperatures with shaking at 180 rpm until the optical density (OD_600_) reached 0.6–0.8 (4 h at 37°C). Protein expression was induced by adding IPTG at a final concentration of 1 mM for various induction periods (4 h at 37°C, 16 h at 18°C and 1 week at 4°C respectively). After IPTG induction, the cells were centrifuged for 30 min at 4°C with a speed of 5,000 x g (Beckman Coulter Inc., United States) to remove the LB medium, and the pellet was resuspended in lysis buffer solution containing 100 mM NaCl, 1 mM EDTA, 1 mM TCEP, 10% glycerol, and 50 mM Tris-HCl at pH 8.5. The resuspended pellet was sonicated for 15 min at 20% amplitude with 2 s intervals between each sonication pulse (Sonics, United States). The lysate was centrifuged for 30 min at 4°C with a speed of 20,000 x g (Beckman Coulter Inc., United States). The supernatant was collected and loaded onto a Ni-NTA column (GE Healthcare, United Kingdom), which was pre-packed and pre-equilibrated with lysis buffer containing 5 mM imidazole. Full-length recombinant espin 1 was eluted stepwise with 10 ml of 50, 100, and 200 mM imidazole gradients, and each eluted fraction was analyzed on a 10% SDS-PAGE gel.

**TABLE 1:** Lists of strains and plasmid used in the study.

Strain	Relevant genotype/phenotype	
DH5α	F^−^ Φ80*lac*ZΔM15Δ(*lac*ZYA-*arg*F) U169 *rec*A1 *end*A1 *hsd*R17 (r_k_^−^,m_k_^+^) *pho*A *sup*E44 *thi*-1 *gyr*A96 *rel*A1 λ^-^	Invitrogen
BL21 (DE3)star	F^−^ *omp*T *hsd*S_B_ (r_B_^−^, m_B_^−^) *gal* *dcm* *rne*131 (DE3)	Invitrogen
pET30a (+)	Km^R,^ *E. coli* expression vector	Novagen

### Other protein sample preparation

Purification of gelsolin protein from bovine serum (Pel-Freez Biologicals, United States) was performed according to the previous study (Kurokawa et al., 1990), which was subjected to dialysis in a buffer consisting of 50 mM Na-acetate, 2 mM MgCl2, 1 mM EGTA, 1.1 mM CaCl2, 0.1 mM MgATP, and 30 mM HEPES at pH 7.5 then and stored at −80°C. Myosin S1 from scallop (*Placopecten magellanicus*) striated adductor muscles was prepared by standard procedure described in (Jung and Craig, 2008; Jung et al., 2008). Purified myosin S1 was further dialyzed in a same buffer used in actin polymerization. Skeletal actin from rabbit skeletal muscle was a generous gift from M. Ikebe laboratory and stored at −80°C before use.

### Size exclusion chromatography

The automatic ÄKTA pure chromatography system and the Superdex 200™ 10/300 GL column (GE Healthcare, United States) were used for SEC. Prior to the sample injection, the Superdex 200™ 10/300 GL column was connected to the automatic ÄKTA pure chromatography system, washed with filtered distilled water and equilibrated with espin 1 buffer containing 50 mM sodium acetate, 2 mM MgCl_2_, 1 mM EGTA, and 30 mM HEPES at pH 7.5. The concentrated sample containing espin 1 was filtered through a 0.2 μm syringe filter and then carefully injected into the 0.5 ml capillary loop of the automated chromatography system, using a 1 ml syringe. SEC was performed at 0.4 ml/min, each 1 ml fraction was collected into a fraction collector and later detected at 280 nm UV wavelength by Ultraviolet (UV) detector in real time. The fractions corresponding to the UV peaks were analyzed using SDS-PAGE.

### Transmission electron microscopy

Skeletal actin was dissolved in a buffer consisting of 50 mM Na-acetate, 2 mM MgCl_2_, 1 mM EGTA, 1.1 mM CaCl_2_, 0.1 mM MgATP, and 30 mM HEPES at pH 7.5 and incubated to polymerize for 30 min at 25°C. This sample (5 μl) was applied to a glow-discharged, carbon-coated grid at 25°C, followed by negative staining with 1% uranyl acetate. The grids were examined using a Technai 10 (FEI, U.S) TEM operated at 100 kV, where the images were recorded using an UltraScan 1000 CCD camera (Gatan, U.S) at magnifications of ×7,000, ×17,000, and ×34,000 i.e., 1.47, 0.62, and 0.62 nm/pixel, respectively. The instrumentation was used at Kangwon Center for Systems Imaging and the images were further compiled and arranged using Adobe Photoshop CS6 (Adobe Inc., U.S). For the Ni-NTA nanogold labeling, based on the affinity of the his_6_-tag for Ni-NTA, 5 nm Ni-NTA nanogold (Nanoprobes, Inc. NY, United States) was used to label the N-terminal his_6_-tag of recombinant espin 1. Samples (F-actin or espin 1) were first loaded onto a carbon coated TEM grid to incubate for 1 min at 25°C. Gold drops (20 μl of gold solution) were applied onto a clean parafilm, and the grids with samples were turned upside down and allowed to float on the gold drops for 1 min incubation at 25°C. After the incubation, the grid was washed three times with buffer drops to remove any non-specific binding. Gold-labeled specimens were then examined from the negative stained TEM images.

### Single particle image processing

Micrographs from TEM at a magnification of ×34,000 (0.32 nm/pixel) were subjected to single-particle analysis using the SPIDER program suite (System for Processing Image Data from Electron microscopy and Related fields; Health Research Inc., Rensselaer, NY, United States) according to procedures described previously (Burgess et al., 2004) in a 100 × 100 pixel window basis, with a globular shape and high frequency. A total of 1,384 particle sets were processed based on the “K-mean clustering” algorithm (Frank et al., 1996; Frank, 2009). All 2D classes were generated using SPIDER.

### F-actin bundling assay (low-speed co-sedimentation)

F-actin, myosin S1, gelsolin, and espin 1 at concentrations of 4 μM, 4 μM, 2 μM, and 1 μM, respectively, were incubated for 30 min at 25°C in a buffer solution containing 50 mM Na-acetate, 2 mM MgCl_2_, 1 mM EGTA, 1.1 mM CaCl_2_, 0.1 mM MgATP, and 30 mM HEPES at pH 7.5. Each mixture of F-actin, gelsolin, and espin 1 was centrifuged for 30 min at 4°C with a speed of 20,000 x g (Hanil, South Korea), followed by removal of the supernatant. An equal amount of the supernatant was compensated with fresh buffer in the pellet. The supernatant and pellet were analyzed using SDS-PAGE and TEM imaging.

## Results

### Purification of espin 1 in different expression temperature

**FIGURE 2:**
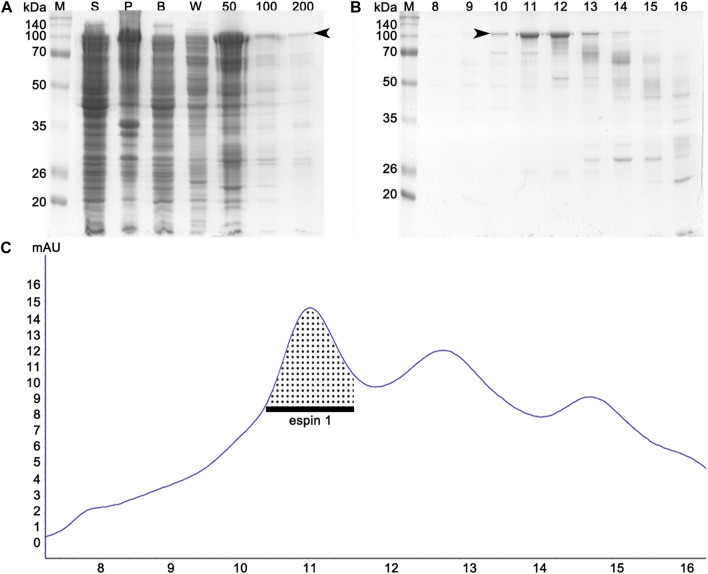
Affinity and size exclusion chromatography profiles of espin 1 purification. **(A)** SDS-PAGE analysis of affinity chromatography. ‘S’ and ‘P’ denote the supernatant and the pellet (obtained from the cell lysate), respectively. Lane B represents a crude solution, while W represents buffer washing after column binding. Imidazole concentrations in the buffer range from 50 to 200 mM. **(B)** SDS-PAGE examination of each separated elution volume i.e., 10–18 ml from SEC. Arrows indicate the espin 1 protein. **(C)** UV profile of SEC which represents the respective sizes and concentration of eluted protein. The dotted area indicates the monomeric portion of the eluted espin 1.

Previous studies have shown that anionic surfactants such as sarkosyl detergents, are essential for heterologous protein expression of human espin 1, which render the synthesized proteins insoluble (Chen et al., 1999). The solubilizing effects of sarkosyl detergents are highly dependent on their concentration; the effects increase the viscosity of the samples, resulting in difficulties in further purification processes (Tao et al., 2010). In this study, a sarkosyl-free approach was used to express soluble espin 1 for the structural analysis. Among the several non-denaturing purification approaches that increase the solubility of recombinant proteins, the low-temperature incubation method was used. To determine the optimized temperature condition on the solubility of recombinant human espin 1, various temperatures ranging from 4°C to 37°C were screened. There were no significant differences in the solubility at 18°C through 37°C induction, but 4°C induction with long period of incubation (i.e., one week) showed notable changes in solubility of espin 1 (Figure 1A). Additional refinement steps were applied using affinity- and size exclusion chromatography; the SEC profile was observed as a single peak and SDS-PAGE results showed a significant major band at 100 kDa, indicating an espin 1 monomer (Figure 2). The functional actin bundling activity of the purified recombinant espin 1 was further verified using TEM (Supplementary Figure S1).

### Actin binding function of espin 1

**FIGURE 3:**
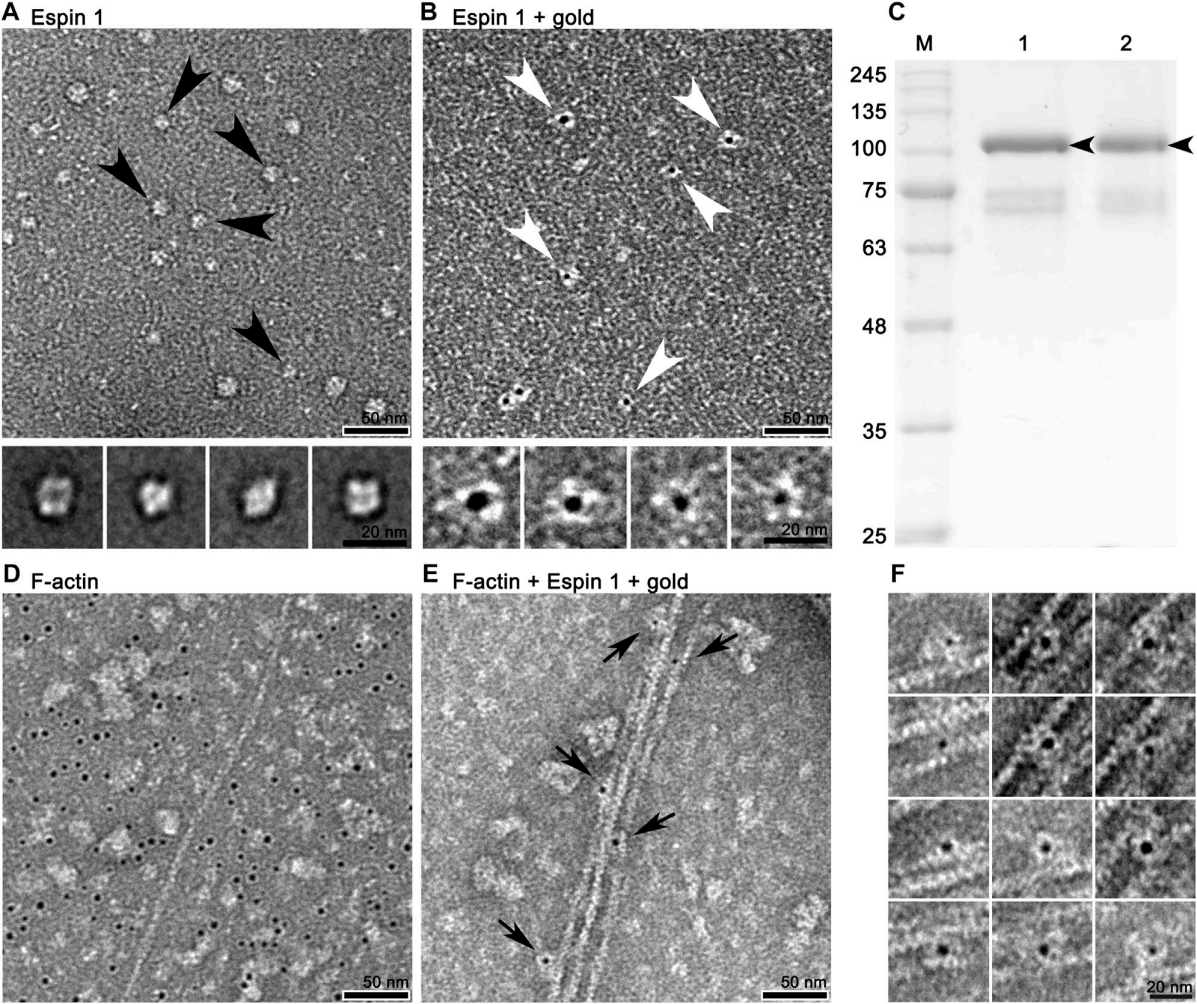
2D appearance of espin 1 visualized by negative staining single particle 2D analysis and functional assay using TEM. **(A)** Negative staining field of purified espin 1 (upper panel) and its 2D class averages (lower panel). Black arrowhead indicates the particles selected for class average. **(B)** Raw micrograph which shows Ni-NTA nanogold labeled espin 1 (upper panel) and focused-view of selected particles (lower panel) as indicated by arrowheads. The gold labeled particle shows 1:1 gold binding ratio indicating monomeric form. Scale bars: 50 nm for upper panel in both A and B; 20 nm for lower panel in both A and **(B) (C)** Non-reducing PAGE of espin 1. Lane 1 indicates reducing control; lane 2 demonstrates non-reducing condition without beta-mercaptoethanol. Black arrows indicate espin 1 band at 100 kDa. **(D,E)** Micrograph of Ni-NTA nanogold treated F-actin **(D)** and actin bundle formation by espin 1 **(E)**. Arrows indicate espin 1 bound to F-actin within the bundles. Note that the localization of Ni-NTA nanogold in the gold-labeled images of espin 1 appears in the central position of the electron density map, possibly due to the position of the his-N-terminal of espin 1. Scale bars: 50 nm. **(F)** Close-up views of the actin bundle areas where Ni-NTA nanogold labeled espin 1 bound. Scale bars: 20 nm.

To visualize and confirm the appearance of purified espin 1, two-dimensional Class average (2D classification) (Figure 3A) and Ni-NTA nanogold labeling (Figure 3B) were done using SPIDER. The 2D class averages of full-length espin 1 suggested that the overall size of the particles was approximately 15 nm, corresponding to the monomeric size of the protein. This was further confirmed by nanogold labeling (Figure 3B; Supplementary Figure S2). Moreover, non-reducing PAGE showed a single band at 100 kDa, implying that purified espin 1 is in monomeric form (Figure 3C). To determine the actin bundling property of espin 1, Ni-NTA nanogold-labeled espin 1 was co-incubated with an actin filament (F-actin) (Figures 3D–F; Supplementary Figure S3). In the experiment, gold labeling was clearly observed along with F-actin, which represents espin 1 monomers binding the actin filaments (Figure 3E). Close-up views of the actin–espin 1 complex indicated that the espin 1 monomers cross-linked the two actin filaments (Figure 3E,F). Taken together, these results suggest that espin 1 monomers can induce actin bundling without multimerization.

### Available espin 1 binding subdomain of F-actin

**FIGURE 4:**
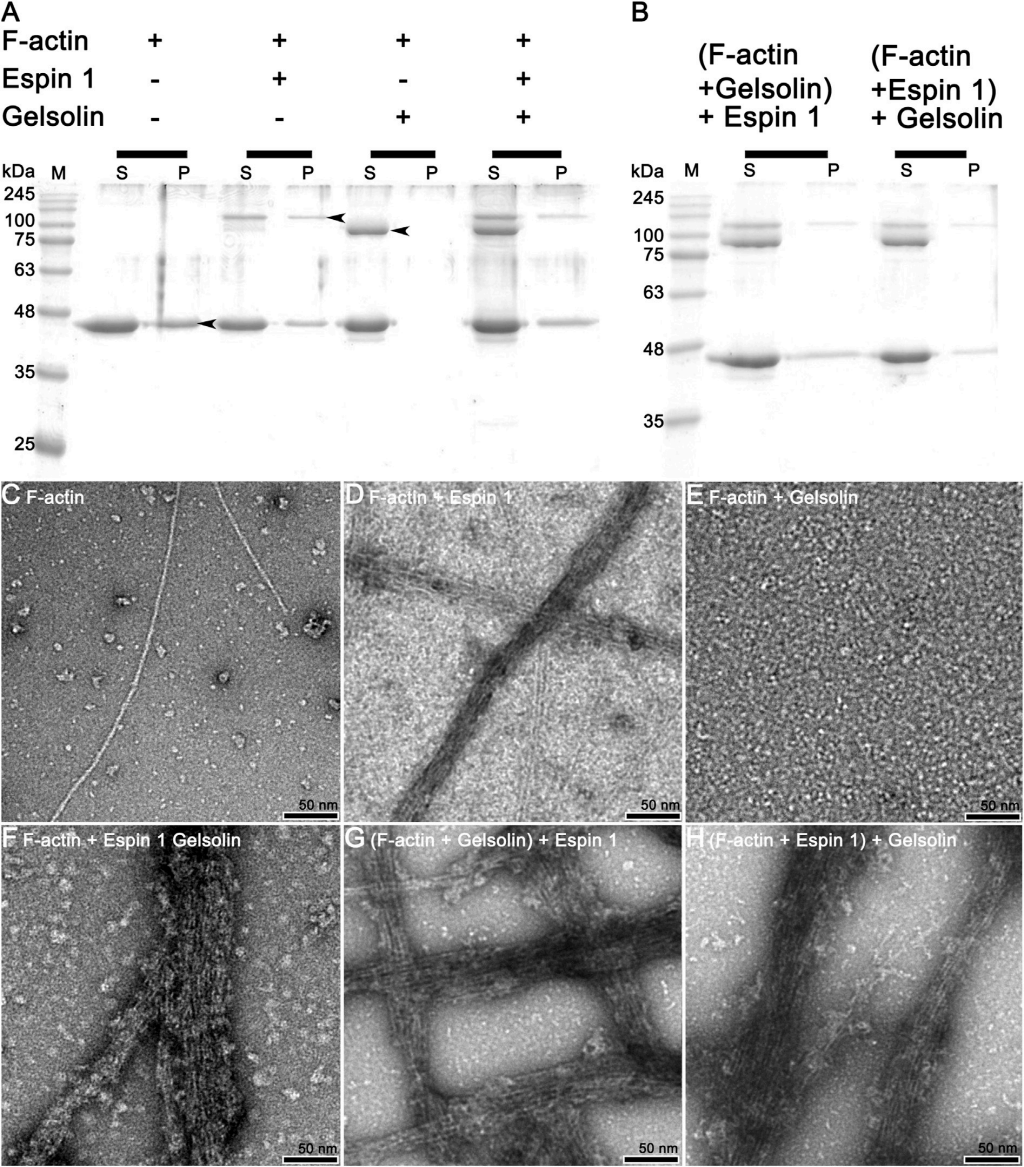
Actin bundling assay (competitive co-sedimentation assay) of espin 1 and gelsolin. **(A,B)** SDS-PAGE results from co-sedimentation assay; **(A)** The general tendency of band migration with each actin binding proteins, gelsolin and espin 1 (Molar ratio of 4:2:1). Lanes S and P represent the supernatant and the pellet respectively. The arrows indicate the relevant to each protein, actin (∼42 kDa), espin 1 (∼100 kDa) and gelsolin (∼84 kDa) respectively. **(B)** Espin 1 and gelsolin treatments in reverse order (i.e., Adding espin 1 to actin-gelsolin mixture (denoted as (F-actin + Gelsolin) +Espin 1) and treating gelsolin to actin–espin 1 mixture (represented as (F-actin + Espin 1) + Gelsolin). M indicates protein marker in kDa. **(C–H)** Electron microscopic analysis of actin-severing and bundling activities of gelsolin and espin 1. Raw micrographs representing F-actin alone **(C)**, F-actin + Espin 1 **(D)**; F-actin + Gelsolin **(E)**; F-actin + Espin 1 + Gelsolin random mixture **(F)**; (F-actin + Gelsolin mixture) + Espin 1 **(G)** and (F-actin + Espin 1 mixture) + Gelsolin **(H)**. Scale bars: 50 nm.

Further studies were conducted to gain more detailed insights into the bundling mechanisms of espin 1. A co-sedimentation assay was performed with gelsolin, which is known to have an actin-severing activity by binding at actin subdomains 1 and 3 (Figure 4) (McLaughlin et al., 1993; Chumnarnsilpa et al., 2009). When espin 1 interacted with actin, both actin and espin 1 bands were found in the pellet, implying actin bundling formation (Figure 4A). In contrast, when gelsolin was treated with actin, the bands relevant to gelsolin were only found in the supernatant portion due to the actin severing activity (Figure 4A). Interestingly, when actin was mixed with both gelsolin and espin 1, gelsolin was found only in the supernatant regardless of the protein addition order, whereas espin 1 was found in pellets with actin (Figures 4A,B). Similar results were observed in the TEM-based structural analysis (Figures 4C–H). First, the actin-bundling activity of espin 1 and the severing effect of gelsolin were confirmed in this experiment (Figures 4D,E). Consistent with the results of previous gel experiments, actin bundling was observed in the actin–espin 1-gelsolin mixture, regardless of the order of addition of each protein (Figures 4F–H). This may be due to the actin binding of espin 1, preventing the access of gelsolin to actin or protecting actin from severing by espin 1 actin bundling.

### Determination of espin 1 binding subdomain of the F-actin

**FIGURE 5:**
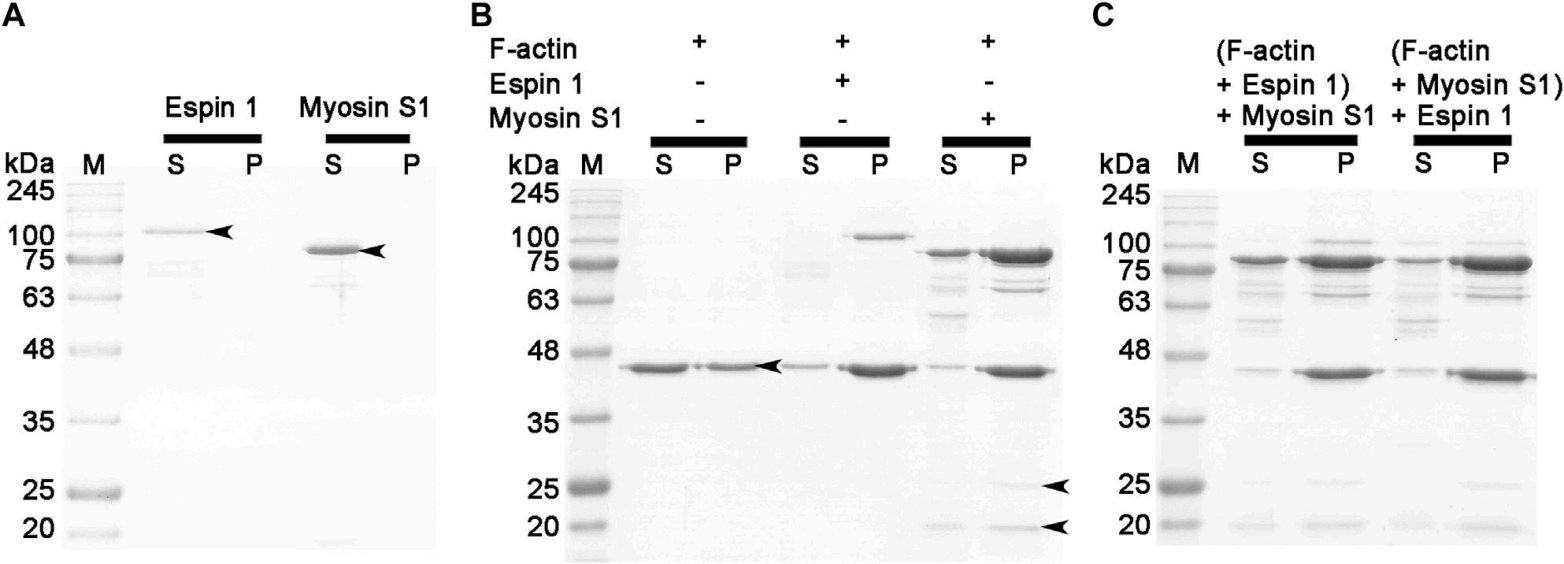
Actin binding assay (competitive co-sedimentation assay) of espin 1 and myosin S1. **(A–C)** SDS-PAGE results derived from co-sedimentation assay. **(A,B)** General tendency of band migration with actin binding proteins, espin 1 and myosin S1 (Molar ratio of 4:4:1). Lanes S and P represent the supernatant and the pellet respectively. The arrows indicate the consistent to each protein, actin (∼42 kDa), espin 1 (∼100 kDa), heavy chain of myosin S1 (∼97 kDa), Essential (∼25 kDa), and Regulatory (∼20 kDa) light chains of myosin S1 respectively. Espin 1 and myosin S1 treatments in reverse order (i.e., Adding myosin S1 to actin–espin 1 mixture (denoted as (F-actin + Espin 1) + Myosin S1) and treating espin 1 to actin-myosin S1 mixture (represented as (F-actin + Myosin S1) + Espin 1). M indicates protein marker in kDa.

To define a more conclusive espin 1 binding subdomain of F-actin, a co-sedimentation assay was performed with myosin S1, which is well-known to bind actin subdomain 1, following a concept similar to the gelsolin treatment experiment (Holmes et al., 2004; Lorenz and Holmes, 2010). Upon centrifugation, both proteins moved to supernatant, indicating a non-polymerized form (Figure 5A). When these proteins were treated with F-actin, the gel band shifted to a pellet band, indicating that each protein was bound to actin filaments (Figure 5B). Furthermore, when F-actin was treated with espin 1 and myosin S1, the SDS-PAGE showed co-localized bands of espin 1, myosin S1, and actin in the pellet, suggesting that these two proteins do not share the same binding site (Figure 5C). Myosin S1 is a well-known actin-binding part of striated muscle myosin containing myosin head which binds to actin subdomain 1 (Holmes et al., 2004). N-terminal ankyrin repeat (AR) of espin 1 is also known to interact with only Tail homology domain (THDI) of myosin 3 and there is no interaction is revealed with C-terminal actin binding domain and myosin head region (Liu et al., 2016). Thus, myosin S1 cannot bind espin 1 but only interact with F-actin indicating one of the espin 1 binding sites of actin is its subdomain 3, but not 1. The gel band thicknesses of espin 1 and myosin S1 showed differences in migration, which may be due to steric hindrance of the actin filament configuration. It is difficult for myosin S1 to bind to the core of the actin bundle after the latter is built by espin 1, and also for myosin S1 decorated actin filaments to form compact actin bundles that interfere with the binding of espin 1.

## Discussion

To date, investigations on espin 1 have focused on understanding the genetic sequences, motifs, domains, actin bundling based molecular studies, stereocilial morphogenesis, and elongation through *in vivo* studies (Bartles et al., 1996; Sekerková et al., 2006b; Zheng et al., 2014; McGrath et al., 2017; Qi et al., 2020). However, the structural organization of espin 1 at the molecular level is still unknown. Moreover, structural analysis requires clear and native conditions, excluding detergent, if possible, but every study of recombinant espin 1 in *E. coli* overexpression has used sarkosyl to dissolve inclusion bodies. For this structure-based study, we controlled and optimized the protein expression rate through low-temperature IPTG induction instead of detergent treatment and successfully purified the soluble form of espin 1 (Figures 1, 2). Using a detergent-free purification of recombinant espin 1 followed by its gel and TEM analyses, this study revealed interesting new results. Based on the simple single-particle analysis (to create 2D class averages of the full-length espin 1 molecule) and Ni-NTA nanogold labeling, it was confirmed that espin 1 formed a monomeric conformation and functioned its actin binding/bundling activity in a monomeric-fashion, *in vitro* (Figure 3). To understand the specific binding subdomain of actin for espin 1, a competitive binding reaction with gelsolin was conducted. As it is well known that actin contains two binding sites for gelsolin (i.e., subdomains 1 and 3), we hypothesized that the actin severing effect of gelsolin can be hindered with espin 1 binding if gelsolin and espin 1 share the binding sites. The actin-severing effect of gelsolin was also shown to be prevented by co-incubation with espin 1 in an order-independent manner (Figure 4). The indirect analytical method for deducing the actin bundling mechanism of espin 1 suggested the possibility that the two proteins share a common subdomain with actin. Since the actin subdomain responsible for gelsolin binding is known, it can be assumed that subdomains 1 and/or 3 could be involved in espin 1 binding. In addition, a subsequent co-sedimentation assay with myosin S1 demonstrated the suggestive evidence that espin 1 binding interface of F-actin is actin subdomain 1 but not 3 (Figure 5). The findings of our study serve as an important methodological platform for further investigations of the actin cytoskeleton. In addition, the findings of this study provide additional understanding of the actin-espin 1 interaction using molecular and structural approaches, and to the best of our knowledge, this is the first study on the espin 1 bundling mechanism. Although it could not confirm specific amino acid residue interactions on the actin and espin 1 binding interface due to negative staining EM resolution limits, indirect tracing results provide suggestive evidence for domain-level actin-espin 1 interactions. More accurate molecular interactions between actin and espin 1 can be determined using high-resolution structural analysis, such as cryo-electron microscopy (cryo-EM). With further research, it is likely that we can determine the mechanism of interaction between actin and espin 1 and provide novel insights for treating various stereocilia-associated disorders, such as hearing loss and vestibular dysfunction.

## Data Availability

The original contributions presented in the study are included in the article/Supplementary Material, further inquiries can be directed to the corresponding authors.

## Abbreviations

DNA, Deoxyribonucleic acid; EGTA, Ethylene glycol-bis (β-aminoethyl ether) -N,N,N′,N′- tetraacetic acid; Espin 1, Ectoplasmic specialization protein 1; F-actin, Filamentous actin; HEPES, 4-(2-hydroxyethyl)-1-piperazineethanesulfonic acid; IPTG, Isopropyl-thiogalactoside; Myosin S1, Myosin sub fragment-1; Ni-NTA, Nickel nitrilotriacetic acid; PAGE, Polyacrylamide gel electrophoresis; SDS, Sodium dodecyl sulfate; SEC, Size exclusion chromatography.

## References

[B1] Bartles J. R. (2000). Parallel actin bundles and their multiple actin-bundling proteins. Curr. Opin. Cell Biol. 12, 72–78. 10.1016/s0955-0674(99)00059-9 10679353 PMC2853926

[B2] Bartles J. R. Wierda A. Zheng L. (1996). Identification and characterization of espin, an actin-binding protein localized to the F-actin-rich junctional plaques of Sertoli cell ectoplasmic specializations. J. Cell Sci. 109, 1229–1239. 10.1242/jcs.109.6.1229 8799813

[B3] Bartles J. R. Zheng L. Li A. Wierda A. Chen B. (1998). Small espin: A third actin-bundling protein and potential forked protein ortholog in brush border microvilli. J. Cell Biol. 143, 107–119. 10.1083/jcb.143.1.107 9763424 PMC2132824

[B4] Burgess S. A. Walker M. L. Thirumurugan K. Trinick J. Knight P. J. (2004). Use of negative stain and single-particle image processing to explore dynamic properties of flexible macromolecules. J. Struct. Biol. 147, 247–258. 10.1016/j.jsb.2004.04.004 15450294

[B5] Chen B. Li A. Wang D. Wang M. Zheng L. Bartles J. R. (1999). Espin contains an additional actin-binding site in its N terminus and is a major actin-bundling protein of the Sertoli cell-spermatid ectoplasmic specialization junctional plaque. Mol. Biol. Cell 10, 4327–4339. 10.1091/mbc.10.12.4327 10588661 PMC25761

[B6] Chumnarnsilpa S. Lee W. L. Nag S. Kannan B. Larsson M. Burtnick L. D. (2009). The crystal structure of the C-terminus of adseverin reveals the actin-binding interface. Proc. Natl. Acad. Sci. U. S. A. 106, 13719–13724. 10.1073/pnas.0812383106 19666531 PMC2720849

[B7] Frank J. (2009). Classification of macromolecular assemblies studied as “single particles”. Q. Rev. Biophys. 23, 281–329. 10.1017/s0033583500005564 2204955

[B8] Frank J. Radermacher M. Penczek P. Zhu J. Li Y. Ladjadj M. (1996). Spider and WEB: Processing and visualization of images in 3D electron microscopy and related fields. J. Struct. Biol. 116, 190–199. 10.1006/jsbi.1996.0030 8742743

[B9] Holmes K. C. Schröder R. R. Sweeney H. L. Houdusse A. (2004). The structure of the rigor complex and its implications for the power stroke. Philos. Trans. R. Soc. Lond. B Biol. Sci. 359, 1819–1828. 10.1098/rstb.2004.1566 15647158 PMC1693467

[B10] Jung H. S. Burgess S. A. Billington N. Colegrave M. Patel H. Chalovich J. M. (2008). Conservation of the regulated structure of folded myosin 2 in species separated by at least 600 million years of independent evolution. Proc. Natl. Acad. Sci. U. S. A. 105 (16), 6022–6026. 10.1073/pnas.0707846105 18413616 PMC2329715

[B11] Jung H. S. Craig R. (2008). Ca2+-induced tropomyosin movement in scallop striated muscle thin filaments. J. Mol. Biol. 14383 (3), 512–519. 10.1016/j.jmb.2008.08.051 PMC2581481 18775725

[B12] Kurokawa H. Fujii W. Ohmi K. Sakurai T. Nonomura Y. (1990). Simple and rapid purification of brevin. Biochem. Biophys. Res. Commun. 168 (2), 451–457. 10.1016/0006-291x(90)92342-w 2334416

[B13] Liu H. Li J. Raval M. H. Yao N. Deng X. Lu Q. (2016). Myosin III-mediated cross-linking and stimulation of actin bundling activity of Espin. eLife 5, e12856. 10.7554/eLife.12856 26785147 PMC4758956

[B14] Loomis P. A. Zheng L. Sekerková G. Changyaleket B. Mugnaini E. Bartles J. R. (2003). Espin cross-links cause the elongation of microvillus-type parallel actin bundles *in vivo* . J. Cell Biol. 163, 1045–1055. 10.1083/jcb.200309093 14657236 PMC2173610

[B15] Lorenz M. Holmes K. C. (2010). The actin-myosin interface. Proc. Natl. Acad. Sci. U. S. A. 107, 12529–12534. 10.1073/pnas.1003604107 20616041 PMC2906587

[B16] Matsudaira P. (1994). Actin crosslinking proteins at the leading edge. Semin. Cell Biol. 5, 165–174. 10.1006/scel.1994.1021 7919230

[B17] McGrath J. Roy P. Perrin B. J. (2017). Stereocilia morphogenesis and maintenance through regulation of actin stability. Semin. Cell Dev. Biol. 65, 88–95. 10.1016/j.semcdb.2016.08.017 27565685 PMC5323431

[B18] McLaughlin P. J. Gooch J. T. Mannherz H. G. Weeds A. G. (1993). Structure of gelsolin segment 1-actin complex and the mechanism of filament severing. Nature 364, 685–692. 10.1038/364685a0 8395021

[B19] Merritt R. C. Manor U. Salles F. T. Grati M. Dose A. C. Unrath W. C. (2012). Myosin IIIB uses an actin-binding motif in its Espin-1 cargo to reach the tips of actin protrusions. Curr. Biol. 22, 320–325. 10.1016/j.cub.2011.12.053 22264607 PMC3288355

[B20] Naz S. Griffith A. J. Riazuddin S. Hampton L. L. Battey J. F. Khan S. N. (2004). Mutations of ESPN cause autosomal recessive deafness and vestibular dysfunction. J. Med. Genet. 41, 591–595. 10.1136/jmg.2004.018523 15286153 PMC1735855

[B21] Pollard T. D. (2016). Actin and actin-binding proteins. Cold Spring Harb. Perspect. Biol. 8, a018226. 10.1101/cshperspect.a018226 26988969 PMC4968159

[B22] Qi J. Zhang L. Tan F. Liu Y. Chu C. Zhu W. (2020). Espin distribution as revealed by super-resolution microscopy of stereocilia. Am. J. Transl. Res. 12, 130–141. 32051742 PMC7013225

[B23] Rotty J. D. Wu C. Bear J. E. (2013). New insights into the regulation and cellular functions of the Arp2/3 complex. Nat. Rev. Mol. Cell Biol. 14, 7–12. 10.1038/nrm3492 23212475

[B24] Salles F. T. Merritt R. C. Manor U. Dougherty G. W. Sousa A. D. Moore J. E. (2009). Myosin IIIa boosts elongation of stereocilia by transporting espin 1 to the plus ends of actin filaments. Nat. Cell Biol. 11, 443–450. 10.1038/ncb1851 19287378 PMC2750890

[B25] Sekerková G. Loomis P. A. Changyaleket B. Zheng L. Eytan R. Chen B. (2003). Novel espin actin-bundling proteins are localized to Purkinje cell dendritic spines and bind the Src homology 3 adapter protein insulin receptor substrate p53. J. Neurosci. 23, 1310–1319. 10.1523/JNEUROSCI.23-04-01310.2003 12598619 PMC2854510

[B26] Sekerková G. Zheng L. Loomis P. A. Changyaleket B. Whitlon D. S. Mugnaini E. (2004). Espins are multifunctional actin cytoskeletal regulatory proteins in the microvilli of chemosensory and mechanosensory cells. J. Neurosci. 24, 5445–5456. 10.1523/JNEUROSCI.1279-04.2004 15190118 PMC2855134

[B27] Sekerková G. Zheng L. Loomis P. A. Mugnaini E. Bartles J. R. (2006a). Espins and the actin cytoskeleton of hair cell stereocilia and sensory cell microvilli. Cell. Mol. Life Sci. 63, 2329–2341. 10.1007/s00018-006-6148-x 16909209 PMC2522319

[B28] Sekerková G. Zheng L. Mugnaini E. Bartles J. R. (2006b). Differential expression of espin isoforms during epithelial morphogenesis, stereociliogenesis and postnatal maturation in the developing inner ear. Dev. Biol. 291, 83–95. 10.1016/j.ydbio.2005.12.021 16413524 PMC2586395

[B29] Small J. V. Stradal T. Vignal E. Rottner K. (2002). The lamellipodium: Where motility begins. Trends Cell Biol. 12, 112–120. 10.1016/s0962-8924(01)02237-1 11859023

[B30] Tao H. Liu W. Simmons B. N. Harris H. K. Cox T. C. Massiah M. A. (2010). Purifying natively folded proteins from inclusion bodies using Sarkosyl, Triton X-100, and CHAPS. BioTechniques 48, 61–64. 10.2144/000113304 20078429

[B31] Zheng L. Beeler D. M. Bartles J. R. (2014). Characterization and regulation of an additional actin-filament-binding site in large isoforms of the stereocilia actin-bundling protein espin. J. Cell Sci. 127, 2208. 10.1242/jcs.174052 PMC6295129 26034062

[B32] Zheng L. Sekerková G. Vranich K. Tilney L. G. Mugnaini E. Bartles J. R. (2000). The deaf jerker mouse has a mutation in the gene encoding the espin actin-bundling proteins of hair cell stereocilia and lacks espins. Cell 102, 377–385. 10.1016/s0092-8674(00)00042-8 10975527 PMC2850054

