# Supplementary figures and images for: Studies of functional properties of espin 1: Its interaction to actin filaments
Source: Front Cell Dev Biol. 2022 Nov 4;10:1022096. doi: 10.3389/fcell.2022.1022096 (PMC9674103; doi:10.3389/fcell.2022.1022096)

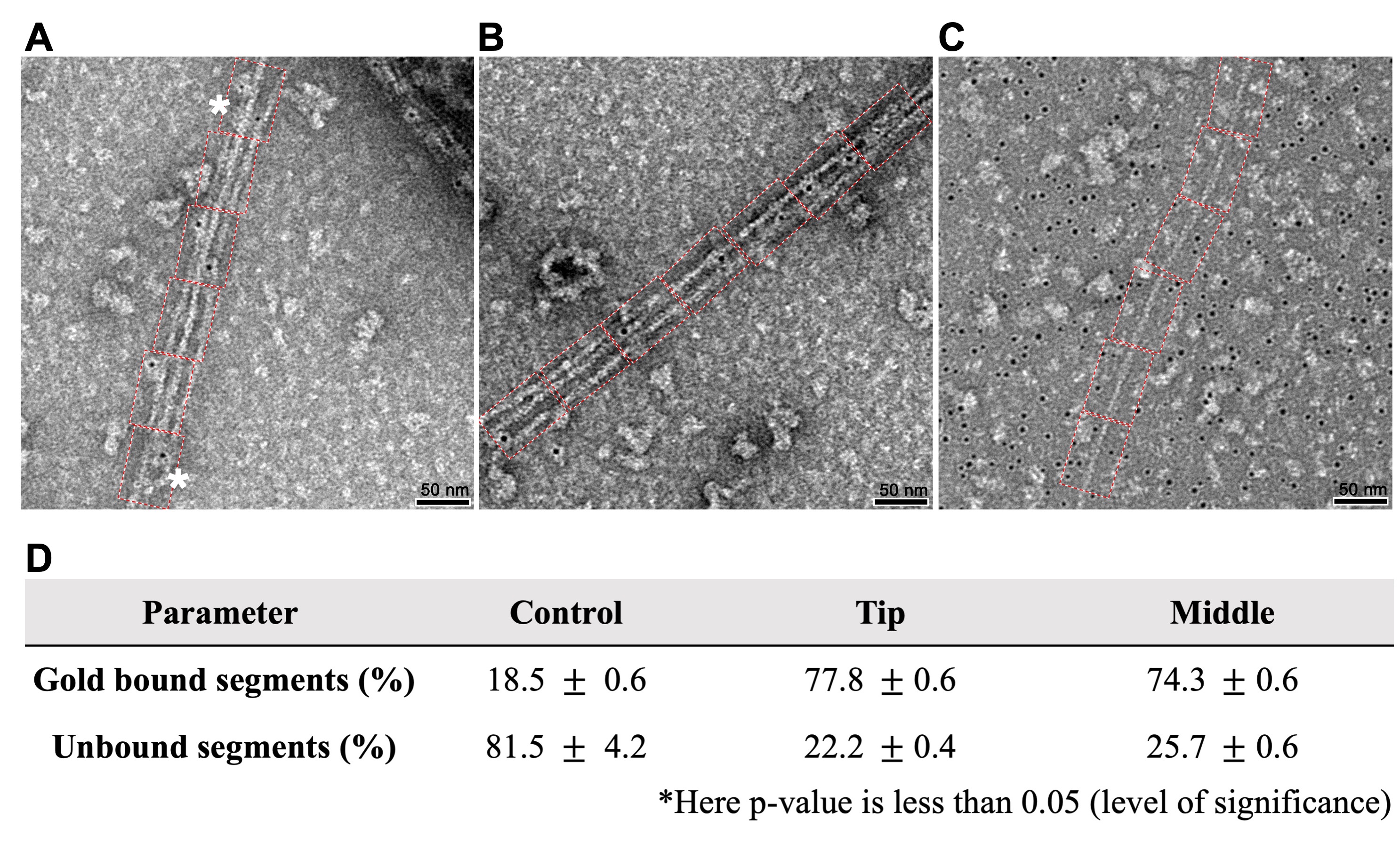

Supplement: Supplementary file 1 [file Image3.JPEG]

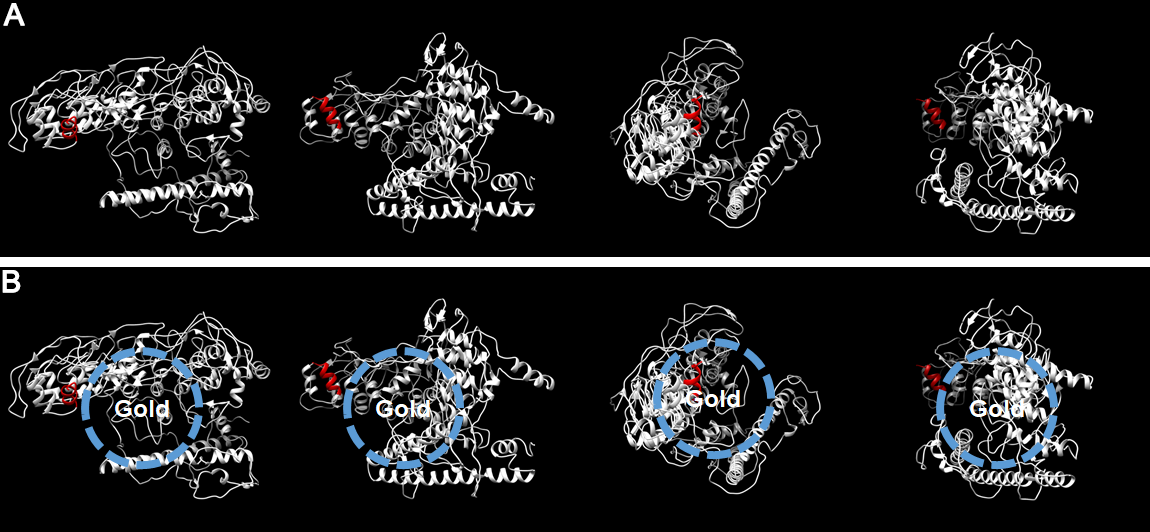

Supplement: Supplementary file 2 [file Image2.TIF]

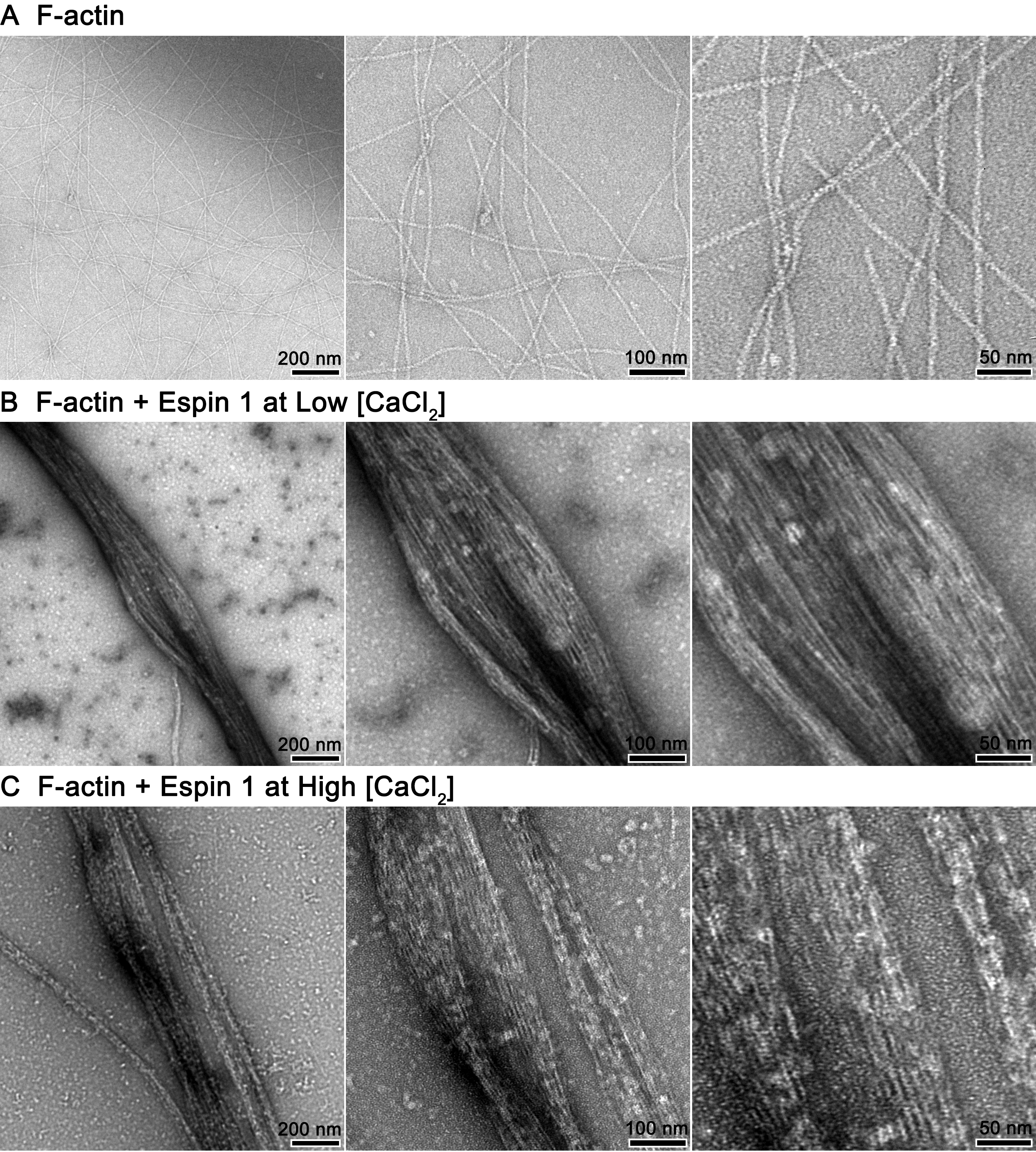

Supplement: Supplementary file 3 [file Image1.TIF]
